# F103. A META-ANALYSIS OF PERSONALITY TRAITS IN DUAL DIAGNOSIS PSYCHOSIS AND SUBSTANCE USE DISORDER

**DOI:** 10.1093/schbul/sby017.634

**Published:** 2018-04-01

**Authors:** Hyeonju Oh, Seon-Kyeong Jang, Kee-Hong Choi

**Affiliations:** 1 Korea University; 2 University of Minnesota, Twin Cities

## Abstract

**Background:**

The comorbidity of substance use and psychotic disorders has been constantly explored in psychosis literature due to its markedly high prevalence and its contribution in debilitating outcomes in various aspects (e.g., lower therapeutic compliance, higher rate of relapse, longer hospitalization, homelessness, and poorer overall functioning). One promising conceptualization of substance use disorder in schizophrenia is understanding shared personality traits such as impulsivity. Thus far, empirical evidences indicated several personality traits as candidate such as impulsivity, neuroticism and anhedonia. However, the accumulated empirical data remains inconsistent across studies. Current study aimed to aggregate data regarding trait personality features in facet level utilizing four-factor UPPS model (Whiteside & Lynam, 2001), four-factor Hierarchical Structural Model (Markon, Krueger, & Watson, 2005), and anhedonia scale for investigating personality traits of impulsivity, neuroticism and anhedonia.

**Methods:**

A systematic literature research was conducted using PubMed, Google Scholar, Scopus, and ProQuest, dissertation database in Proquest, and hand searches from reference lists of identified articles. Our analysis covers all studies published up to May 2017. The electronic database research resulted in an initial pool of 110 studies in total, and 12 studies remains for current meta-analysis after screening by inclusion/exclusion criteria and removing duplicate studies. Two authors (SKJ and HJO) independently coded data, and all authors cross-checked to ensure the reliability of coded data to reach consensus. Effect-size estimates were calculated based on means and standard deviations of psychotic disorder only group (PSD) and dual diagnosis group (DD) on personality scales using R package metafor. Specifically, Hedges’ g was derived with a random-effect model, allowing unbiased effect sizes adjusted for different sample sizes that helps to infer population-level estimates. To examine potential confounding factors, independent two-sample t-tests were conducted between DD and PSD group for any significant differences in demographic and clinical characteristics. Fail-Safe N test was utilized to address publication bias of the current meta-analysis, and inconsistency of data was assessed using heterogeneity measure (I2). The methodological quality assessment of included studies was created by using an adapted version of Agency for Healthcare Research and Quality (AHRQ) tool for observational studies.

**Results:**

There were no statistically significant baseline differences in demographic and clinical characteristics between DD and PSD group. The results indicate that DD patients exhibited significantly higher scores on negative urgency, low premeditation, and sensation seeking compared to PSD within the UPPS model. However, low perseveration did not differ between two groups. Within the HS model, unconscientious disinhibition was significantly higher in DD compared to PSD, but not negative affect, disagreeable disinhibition, and positive affect. Lastly, trait anhedonia score was not significantly different between groups.

**Discussion:**

Despite a limited number of available studies, our meta-analysis allowed to specify the personality trait in facet level of dual diagnosis patients compared to patients without substance use disorder. We conclude that the personality trait of DD patients may lead to the employment of urgent emotional regulation strategies (i.e. substance use) to alleviate negative emotion. More effective emotion regulations strategies (e.g., mindfulness, emotion tolerance skills, etc) might need to be integrated for treatment for DD patients.

